# Generation of therapeutic antisera for emerging viral infections

**DOI:** 10.1038/s41541-018-0082-4

**Published:** 2018-10-05

**Authors:** Rebecca Schmidt, Lea C. Beltzig, Bevan Sawatsky, Olga Dolnik, Erik Dietzel, Verena Krähling, Asisa Volz, Gerd Sutter, Stephan Becker, Veronika von Messling

**Affiliations:** 1 Veterinary Medicine Division, Paul-Ehrlich-Institut, Langen, Germany; 2 Thematic Translational Unit Emerging Infections, German Center of Infection Research (DZIF), Marburg - Langen - Munich, Germany; 3 Institute of Virology, Philipps University Marburg, Marburg, Germany; 4 Institute for Infectious Diseases and Zoonoses, Ludwig-Maximilians-University, Munich, Germany

## Abstract

The recent Ebola virus outbreak has highlighted the therapeutic potential of antisera and renewed interest in this treatment approach. While human convalescent sera may not be readily available in the early stages of an outbreak, antisera of animal origin can be produced in a short time frame. Here, we compared adjuvanted virus-like particles (VLP) with recombinant modified vaccinia virus Ankara and vesicular stomatitis virus (VSV), both expressing the Ebola virus antigens. The neutralizing antibody titers of rabbits immunized with adjuvanted VLPs were similar to those immunized with the replication-competent VSV, indicating that presentation of the antigen in its native conformation rather than de novo antigen expression is essential for production of functional antibodies. This approach also yielded high-titer antisera against Nipah virus glycoproteins, illustrating that it is transferable to other virus families. Multiple-step immunoglobulin G purification using a two-step 20–40% ammonium sulfate precipitation followed by protein A affinity chromatography resulted in 90% recovery of functionality and sustained in vivo stability. Adjuvanted VLP-based immunization strategies are thus a promising approach for the rapid generation of therapeutic antisera against emerging infections.

## Anti-serum: Rapid generation for viral outbreaks

Passive immunity through the transfer of anti-serum represents the earliest clinical application of antibodies and is still widely used to this day in the form of anti-venoms. Veronika von Messling and colleagues at the Paul Ehrlich Institute investigate the potential of generating neutralizing anti-serum to the emerging viruses Ebola and Nipah. The authors compare different vaccination platforms in mice and rabbits and find that following multiple vaccine challenges, neutralizing antibody titers equivalent to that seen in convalescent patients could be obtained. Purification of the IgG fraction and processing into F(ab’)_2_ fragments has the potential to significantly reduce xeno-responses yet the authors find that neutralizing capacity is largely retained albeit at the cost of a shorter *in vivo* half-life. These findings offer the hope of rapidly generating large quantities of neutralizing anti-serum that could be used in a viral outbreak scenario.

## Introduction

Outbreaks of emerging viruses occur with worrying regularity, and while some are quickly controlled locally, others become public health events of global concern. Among the pathogens involved, viruses that can be transmitted directly and cause severe disease with high mortality such as filovirus and henipavirus are considered World Health Organization (WHO) priority pathogens. Until the recent West African Ebola virus (EBOV) epidemic, which resulted in 28,616 confirmed cases and 11,310 deaths,^1,2^ EBOV outbreaks were generally small and rapidly contained.^3,4^ The same holds true for Nipah (NiV) and Hendra (HeV) viruses, which so far have mainly caused local clusters of infection,^5,6^ or small outbreaks as the one currently ongoing in India.^7^ However, the first NiV outbreak in Malaysia and Singapore in 1998–1999 resulted in 276 documented infections with a case fatality rate of approximately 40%,^8,9^ illustrating the challenges associated with predicting and preparing for such events. Since licensed therapies or vaccines are often not immediately available, patient isolation, supportive care, and contact tracing are the main approaches used to control these outbreaks.^10,11^ There is thus a renewed interest in therapeutic approaches such as convalescent sera or hyperimmune sera of animal origin, which can be rapidly available.

Passive antibody treatment with whole blood or plasma from convalescent patients has been used as experimental therapy during filovirus outbreaks since 1967,^12,13^ with variable results. Whole blood from convalescent donors was given as a post-exposure treatment during the 1995 Kikwit outbreak with seven of eight patients surviving the infection.^14^ However, post-exposure treatment of nonhuman primates with whole blood from convalescent donors did not replicate the successes seen in human patients.^15^ More recently, 84 patients treated with convalescent plasma failed to show significant improvement in survival during the 2014 West African outbreak,^16^ but definitive interpretation of these data is difficult because the antibody titers and virus-neutralizing activity of the convalescent plasma was not reported. Post-exposure prophylaxis studies in EBOV-infected nonhuman primates using high-titer purified immunoglobulin G (IgG) from convalescent macaques is fully protective against subsequent challenge,^17^ indicating that functional antibody titers may be the determining factor. Neutralizing antibodies are also important mediators of protection against NiV.^18^ Post-exposure passive transfer of sera from hamsters immunized with either vaccinia or vesicular stomatitis viruses (VSVs) expressing the NiV-G protein provides protection from lethal virus challenge in a hamster model,^19,20^ as do murine monoclonal antibodies that bind either NiV-F or NiV-G,^21^ and a human monoclonal antibody with neutralizing activity against the related Hendra virus G protein in ferrets.^22^

In general, passive immunization has proven to be beneficial for numerous acute infections and is still commonly used as emergency treatment against life-threatening diseases. Human IgG preparations are the gold standard for rabies^23^ and tetanus^24^ post-exposure prophylaxis, and are available as emergency treatment for anthrax.^25^ Pooled human IgG for intravenous use (IVIG) is used as a prophylactic and post-exposure treatment against hepatitis B,^26^ and is given to patients with complications during measles infection or to those that are at risk for severe disease.^26^

While human convalescent sera may not be readily available in outbreak situations, antisera of animal origin can be produced in a short time frame. Animal-origin purified IgG^27^ and IgG fragment preparations are still routinely used as antivenoms, and even though immunization of horses with inactivated EBOV failed to yield effective therapeutic antisera,^28^ equine hyperimmune sera produced using EBOV virus-like particles (VLPs) conferred protection against lethal challenge in rodents.^29^ Here we compared different immunization platforms for the rapid induction of high functional antibody titers against EBOV and NiV, and optimized the subsequent IgG purification process.

## Results

### Squalene-containing adjuvants enhance EBOV VLP-induced total and functional antibody titers

**Fig. 1:**
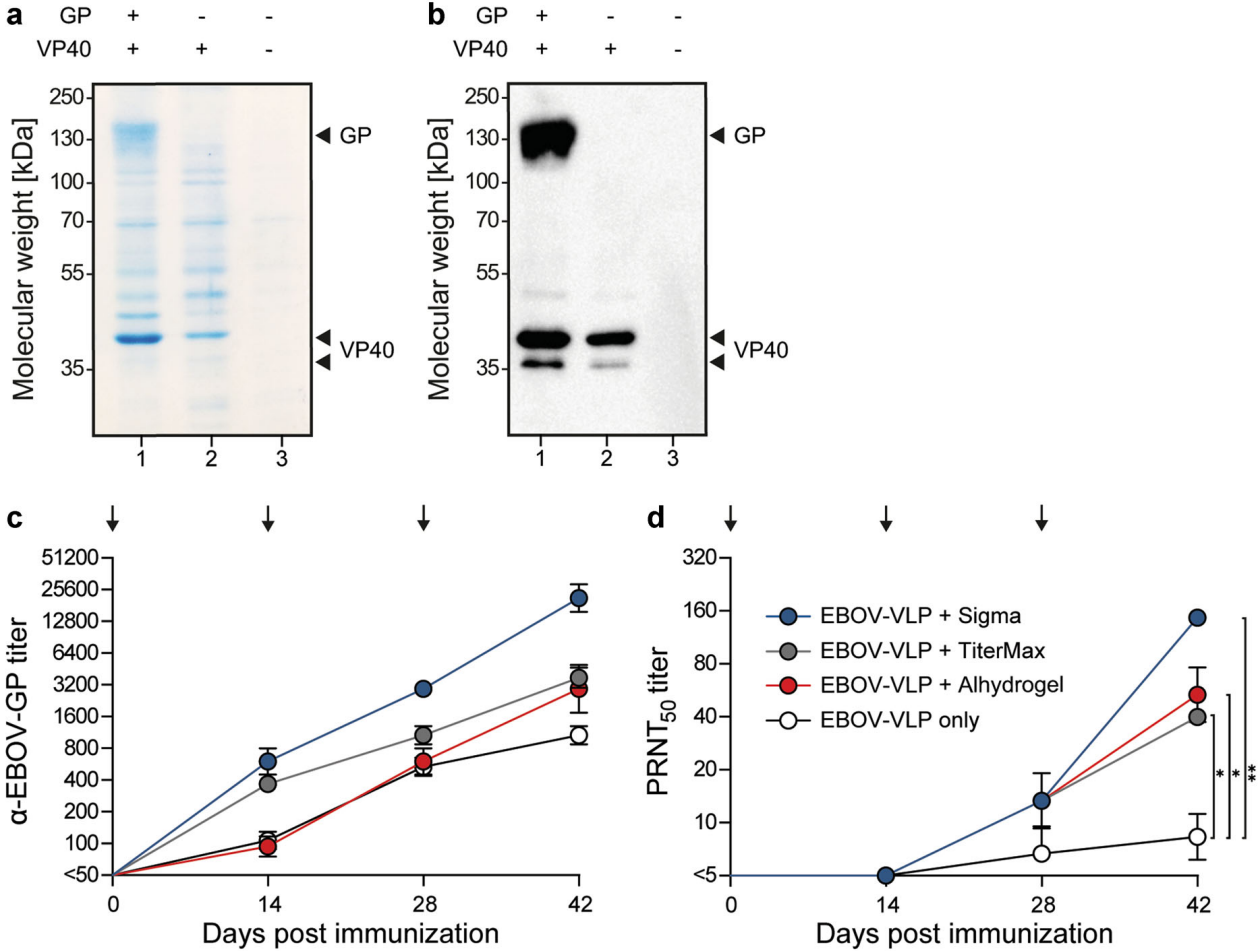
Ebola VLP production and antibody response kinetics. **a**, **b** VLP preparations analyzed after transfection of HEK-293 cells, harvest of the VLP-containing supernatant, purification, and concentration by ultracentrifugation through a sucrose cushion. Samples were separated on SDS-PAGE gels and proteins were **a** stained with Coomassie blue or **b** transferred to PVDF membranes. Blots were stained with a polyclonal goat antiserum against EBOV. Lane 1: 10 µg VLP sample containing Ebola VP40 and GP; lane 2: 10 µg VLP sample containing only Ebola VP40; and lane 3: 10 µl negative control (not transfected). All blots were derived from the same experiment and were processed in parallel. **c**, **d** Mice were immunized i.m. with 10 µg of VLPs alone or in combination with either Sigma Adjuvant System, TiterMax Gold, or Alhydrogel, and boosted 2 and 4 weeks after the first immunization. Final serum samples were collected 2 weeks after the second boost. The total antibody response against recombinant EBOV-GP **c** and neutralizing antibody response against VSVΔG/EBOV-GP **d** are shown. Antibodies against EBOV-GP are reported as reciprocal serum endpoint titers using IPMA and neutralizing antibodies were assessed by the 50% serum neutralization capacity (PRNT_50_). Symbols represent the mean of each group (*n* = 3), and error bars indicate the standard error of the mean. The *Y*-axis begins at the detection limit of the respective assays. Statistical significance is indicated by *p < 0.05 and **p < 0.01

VLPs are an effective platform for the display of antigens in their native conformation.^30,31^ To assess their ability for induction of high functional antibody titers, we produced EBOV VLPs by co-transfecting human embyonic kidney-293 (HEK-293) cells with plasmids encoding for the EBOV Zaire strain Mayinga matrix protein (VP40) and the full-length membrane-anchored glycoprotein (GP).^31^ After 48 h, the supernatant was harvested, purified, and concentrated via ultracentrifugation. The resulting EBOV VLP preparation contained two major proteins migrating in Coomassie-stained gels at 130 kDa and around 40 kDa (Fig. 1a). Western blot analysis identified the distinct band at 130 kDa as EBOV-GP1, the large cleavage fragment of EBOV-GP,^31,32^ and two bands between 35 and 55 kDa as two isoforms of EBOV-VP40 due to a second upstream in-frame start codon^33^ (Fig. 1b).

To evaluate the effect of adjuvants on total and functional antibody responses, groups of mice were immunized intramuscularly with 10 μg EBOV VLP alone, or in combination with the squalene-containing water-in-oil Sigma adjuvant, the oil-in-water TiterMax adjuvant, or 2% Alhydrogel, all of which are routinely used for animal immunizations, and boosted 2 and 4 weeks later. Serum was collected 2 weeks after each immunization and kinetics of total and neutralizing antibody responses were followed. First antibodies against EBOV-GP were detected after 2 weeks, with approximately 10-fold higher titers in the Sigma and TiterMax adjuvant groups. The titers in all groups gradually increased over the course of the immunizations, but while the final titers induced by non-adjuvanted EBOV VLPs were similar to those in the Alhydrogel-adjuvanted and TiterMax-adjuvanted groups, Sigma adjuvant resulted in 10-fold higher final titers (Fig. 1c). Neutralizing antibody titers were determined by plaque reduction neutralization test (PRNT) with the recombinant VSV that expresses the GP of the closely related EBOV Zaire strain Kikwit (VSVΔG/EBOV-GP).^34^ First neutralizing antibodies were detected after the second immunization, but a robust increase was only observed in the adjuvanted groups, with a 5-fold increase in the TiterMax-adjuvanted and Alhydrogel-adjuvanted groups and a 20-fold increase in the Sigma adjuvant group (Fig. 1d), leading us to choose Sigma adjuvant for all further experiments.

### Induction of robust neutralizing antibody titers requires several immunizations

**Fig. 2:**
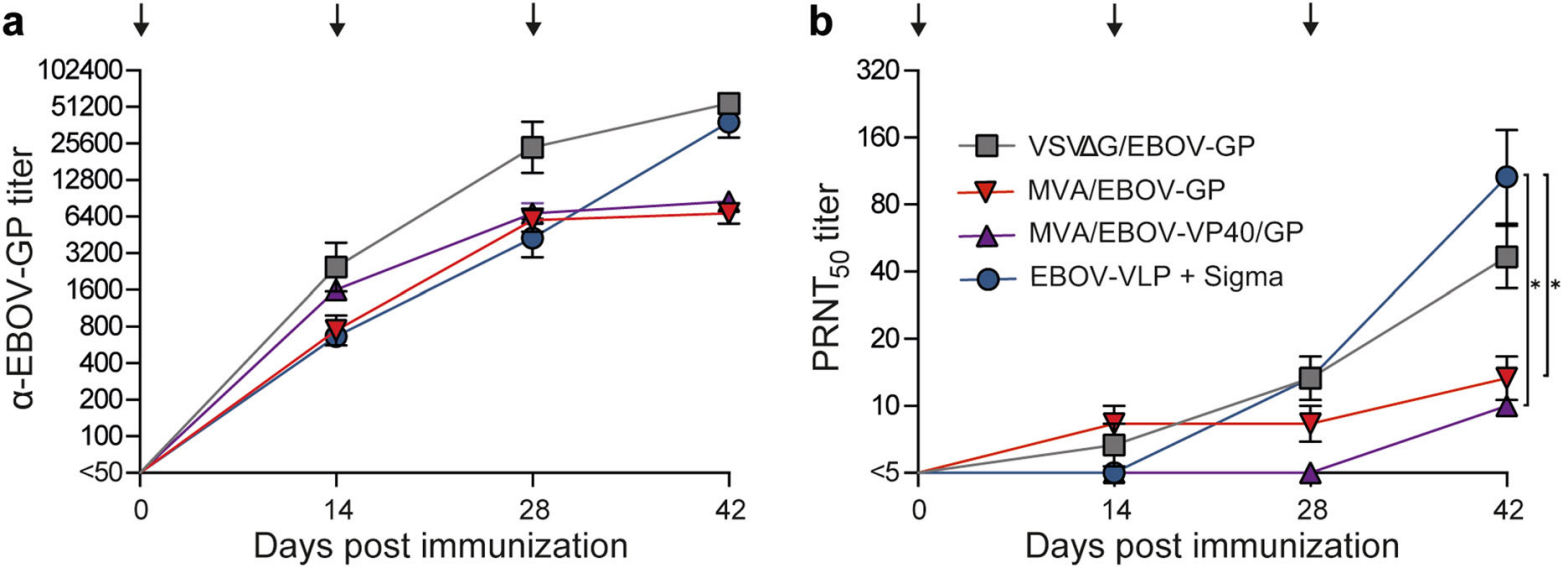
Comparison of total and neutralizing antibody responses against EBOV-GP induced in mice using different immunization approaches. Animals were immunized i.m. with 2 × 10^5^ PFU VSVΔG/EBOV-GP, 1.5 × 10^8^ FFU of either MVA/EBOV-VP40/GP or MVA/EBOV-GP, or 10 µg of EBOV VLPs in combination with Sigma adjuvant, and boosted 2 and 4 weeks later before final serum was collected 2 weeks after the second boost. The total antibody response against recombinant EBOV-GP **a** and neutralizing antibody response against VSVΔG/EBOV-GP **b** are shown. Antibodies against EBOV-GP are reported as reciprocal serum endpoint titers using IPMA and neutralizing antibodies were assessed by the 50% serum neutralization capacity (PRNT_50_). Symbols represent the mean of each group (*n* = 3), and error bars indicate the standard error of the mean. The *Y*-axis begins at the detection limit of the respective assays. Statistical significance is indicated by *p < 0.05

VLPs lack the ability for de novo protein production. To determine the importance of this aspect for the induction of functional antibodies, we compared adjuvanted EBOV VLPs with vector vaccines based on the replication-deficient modified vaccinia Ankara virus (MVA) that expresses either the GP protein of the Zaire EBOV Guéckédou strain alone or in combination with VP40 (unpublished data), and the replication-competent VSVΔG/EBOV-GP.^34^ Due to the around 80% amino acid sequence identity and strong structural and functional conservation among Zaire EBOV-GP proteins, the induced immune responses are cross-reactive within the species.^35,36^ As before, mice were immunized three times in 2-week intervals. Total antibody titers against EBOV-GP increased rapidly after initial immunization in all groups, but in contrast to the adjuvanted EBOV VLPs and the VSVΔG/EBOV-GP, there was no further increase after the third immunization with the MVA-based viral vectors (Fig. 2a). Neutralizing antibodies were detected in MVA/EBOV-GP-immunized groups after the first and MVA/EBOV-VP40/GP-immunized groups after the second immunization and slightly increased to yield final titers around 10 (Fig. 2b). The first neutralizing antibodies in VSVΔG/EBOV-GP animals were also detected after the first immunization with final titers reaching 40 (Fig. 2b). Even though the neutralizing antibodies were again only detected after the first boost in the group immunized with adjuvanted EBOV VLPs, titers ultimately reached levels around 100 (Fig. 2b), illustrating that de novo protein expression is not required for efficient induction of functional antibodies.

### Immunization with adjuvanted EBOV VLPs induces high neutralizing antibody titers in rabbits

To generate larger volume of antisera for optimization of the purification process and to assess the scalability of antisera production, we repeated the immunization protocol in rabbits. Animals were immunized with the adjuvanted EBOV VLPs, MVA/EBOV-GP, or VSVΔG/EBOV-GP, and boosted 2 and 4 weeks later. Serum was collected 1 week after the second boost. Total anti-EBOV-GP antibody titers (Table 1, bottom row) and neutralizing antibody levels against VSVΔG/EBOV-GP (Table 2, bottom row) were slightly higher than the titers seen in mice, validating the immunization scheme in a different species.

**Table 1:** Comparison of total antibody responses against EBOV and EBOV-GP induced in rabbits using different antigen expression approaches

	VSVΔG/EBOV-GP (*n* = 1)	MVA/EBOV-GP (*n* = 2)	300 µg VLPs + adjuvant (*n* = 2)
α-EBOV endpoint titer (log_2_) (ELISA)	16	14	15
α-EBOV-GP endpoint titer (log_2_) (IPMA)	16	15	16

**Table 2:** Comparison of neutralizing antibody responses against EBOV and VSVΔG/EBOV-GP induced in rabbits using different antigen expression approaches

	VSVΔG/EBOV-GP (*n* = 1)	MVA/EBOV-GP (*n* = 2)	300 µg VLPs + adjuvant (*n* = 2)
α-EBOV endpoint titer (log_2_) (VNT)	10	7	10
α- VSVΔG/EBOV-GP titer (log_2_)(PRNT_50_)	8	5	8

To put the titers obtained after immunization of rabbits in a clinically relevant context, total anti-EBOV antibodies were quantified in a validated whole-virion EBOV antibody capture enzyme-linked immunosorbent assay (ELISA), used to assess the antibody responses in survivors of EBOV virus disease during the West African outbreak,^37^ as well as a virus neutralization assay (VNT) with EBOV Zaire strain Mayinga used to assess antibody responses in VSVΔG/EBOV-GP vaccinated volunteers using the same assay.^38^ The optical density (OD) values of rabbit sera tested in the validated whole-virion EBOV ELISA ranged from 3.5 to 4.1 and were thus comparable to high-titer convalescent sera after patients had cleared the virus.^37^ Endpoint titrations revealed comparable total antibody titers against EBOV and EBOV-GP (Table 1). The neutralizing antibody titers assessed in VNT with EBOV were for MVA/EBOV-GP rabbit sera more than 10-fold and for VSVΔG/EBOV-GP and VLP rabbit sera more than 75-fold higher than the average geometric mean neutralizing endpoint titers of 16 (2^4^) or 11 (2^3.5^) in vaccines at day 28 or 180 (Table 2), indicating that titers of the rabbit hyperimmune sera are within the therapeutic range.^38^ While more systematic bridging studies would be required to directly correlate our in-house with the official diagnostic assays, the similar relative proportions of the titers induced by the different immunization platforms indicates their suitability for initial analyses.

### VLP-based antisera production is applicable to other emerging viral infections

**Fig. 3:**
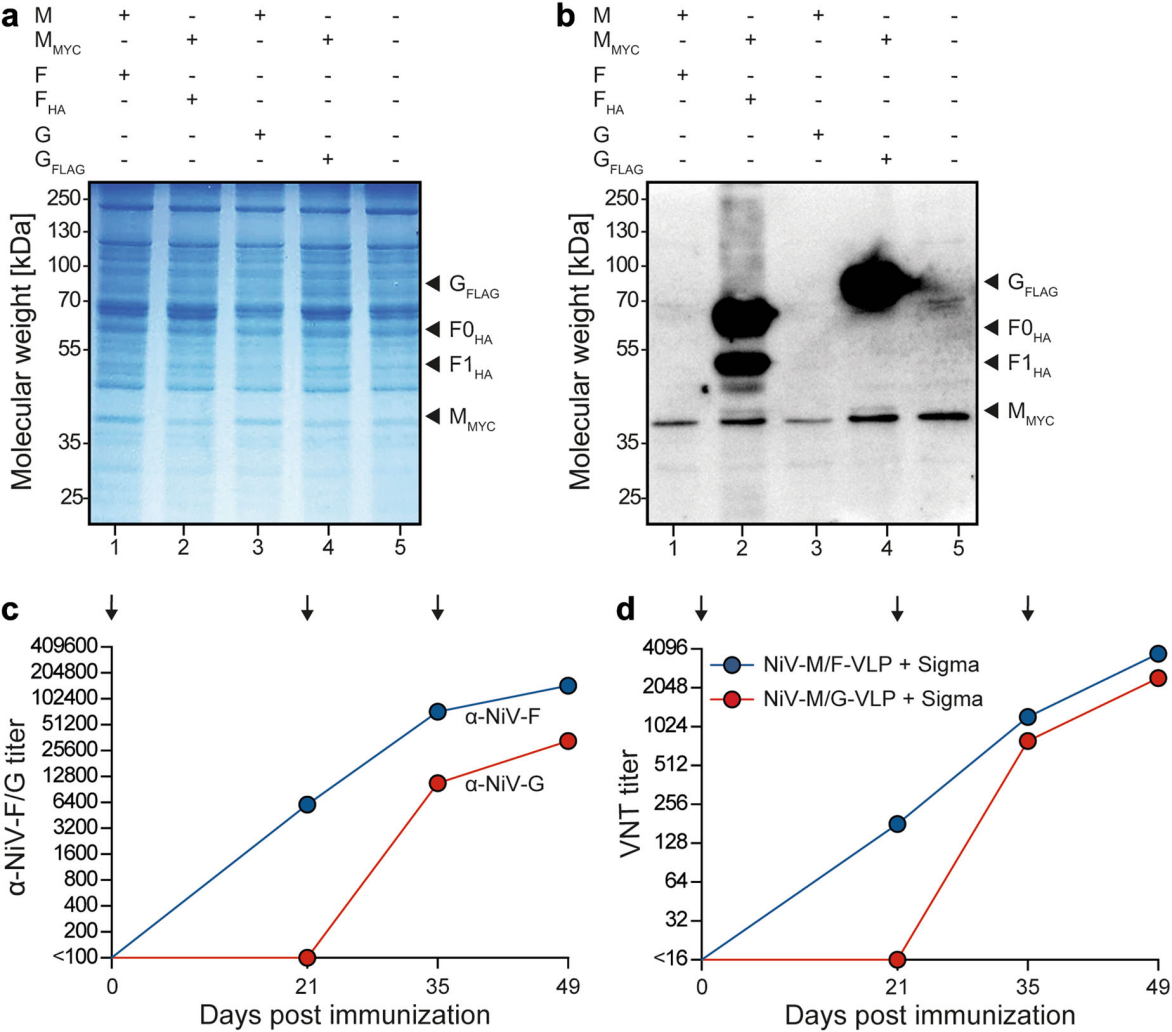
Nipah VLP production and antibody response kinetics. **a**, **b** VLP preparations analyzed after transfection of HEK-293 cells with NiV-M and NiV-F protein or NiV-M and NiV-G protein with or without epitope tags, harvest of the VLP-containing supernatant, purification, and concentration by ultracentrifugation through a sucrose cushion. Samples were separated on SDS-PAGE gels and proteins were **a** stained with Coomassie blue or **b** transferred to PVDF membranes. Blots were stained with monoclonal antibodies against the c-myc, HA, and FLAG tags. Lane 1: 3 µg VLP sample containing NiV-M and NiV-F; lane 2: 3 µg VLP sample containing NiV-M_myc_ and NiV-F_HA_; lane 3: 3 µg VLP sample containing NiV-M and NiV-G; lane 4: 3 µg VLP sample containing NiV-M_myc_ and NiV-G_FLAG_; lane 5: negative control (not transfected). All blots were derived from the same experiment and were processed in parallel. **c**, **d** Rabbits were immunized i.m. with 300 µg of NiV VLPs containing either M and F or M and G protein and boosted 3 and 5 weeks after the first immunization. Final serum samples were collected 2 weeks after the second boost. The total antibody response against recombinant NiV-F or NiV-G proteins (**c**) and neutralizing antibody response against NiV strain Malaysia (**d**) are shown. Total antibodies against NiV-F or NiV-G and neutralizing antibodies are reported as reciprocal serum endpoint titers using IPMA or VNT. Symbols represent the geometric mean of each group (*n* = 2). The *Y*-axis begins at the detection limit of the respective assays

Immunization with recombinant viruses or VLPs containing the NiV fusion (F) and attachment (G) surface GPs is sufficient to elicit a protective immune response,^20,39,40^ and there is considerable cross-reactivity with different NiV and even Hendra virus strains.^41^ To evaluate the applicability of the VLP-based immunization approach for the production of high-titer antisera against other emerging viruses, NiV VLPs were generated by co-transfecting HEK-293T cells with plasmids encoding the NiV matrix protein (M) in combination with either the F or G protein of the NiV Malaysia strain.^42^ After 48 h, supernatant was harvested, purified and concentrated via ultracentrifugation. The resulting NiV-M/G and NiV-M/F VLP preparations showed no distinct bands migrating in Coomassie-stained sodium dodecyl sulfate-polyacrylamide gel electrophoresis (SDS-PAGE) gels (Fig. 3a), but bands corresponding to NiV-M (44 kDa), NiV-G (68 kDa), and two bands representing uncleaved NiV-F_0_ and cleaved NiV-F_1_ (approximately 60 and 48 kDa, respectively) could be readily detected by Western blot analysis (Fig. 3b).^43^

To assess the total and functional antibody responses induced by immunization with the respective NiV GP, two rabbits were immunized intramuscularly with 300 μg NiV-M/F or NiV-M/G VLPs in combination with the Sigma adjuvant. Animals were boosted 3 and 5 weeks later and serum was collected before each immunization and 2 weeks after the second boost to follow the kinetics of the total and neutralizing antibody responses.

Antibodies against NiV-F in rabbits immunized with NiV-M/F VLPs were detected after the first immunization, while induction of antibodies against NiV-G required two immunizations with NiV-M/G VLPs (Fig. 3c). Subsequent immunizations increased total antibody titers, and titers of NiV-F antibodies ultimately reached levels approximately two times higher than NiV-G antibodies (Fig. 3c). Consistent with these findings, neutralizing antibody titers against the Malaysia strain were found in the NiV-M/F VLP group after the first immunization, while neutralizing antibodies against NiV-M/G were first detected after the second immunization, and these titers increased in both groups of animals after the third and fourth immunizations (Fig. 3d). The neutralizing titers against the NiV-F and NiV-G glycoproteins (approx. 3000) were well above the level of neutralizing activity (>160 neutralizing units/mL) that protects against lethal NiV challenge in passive transfer studies,^19,20^ which suggests that our rabbit anti-NiV GP sera would also provide a similar degree of protection.

### Multi-step purification process yields IgG and F(ab′)_2_ samples with high recovery of neutralizing activity

**Fig. 4:**
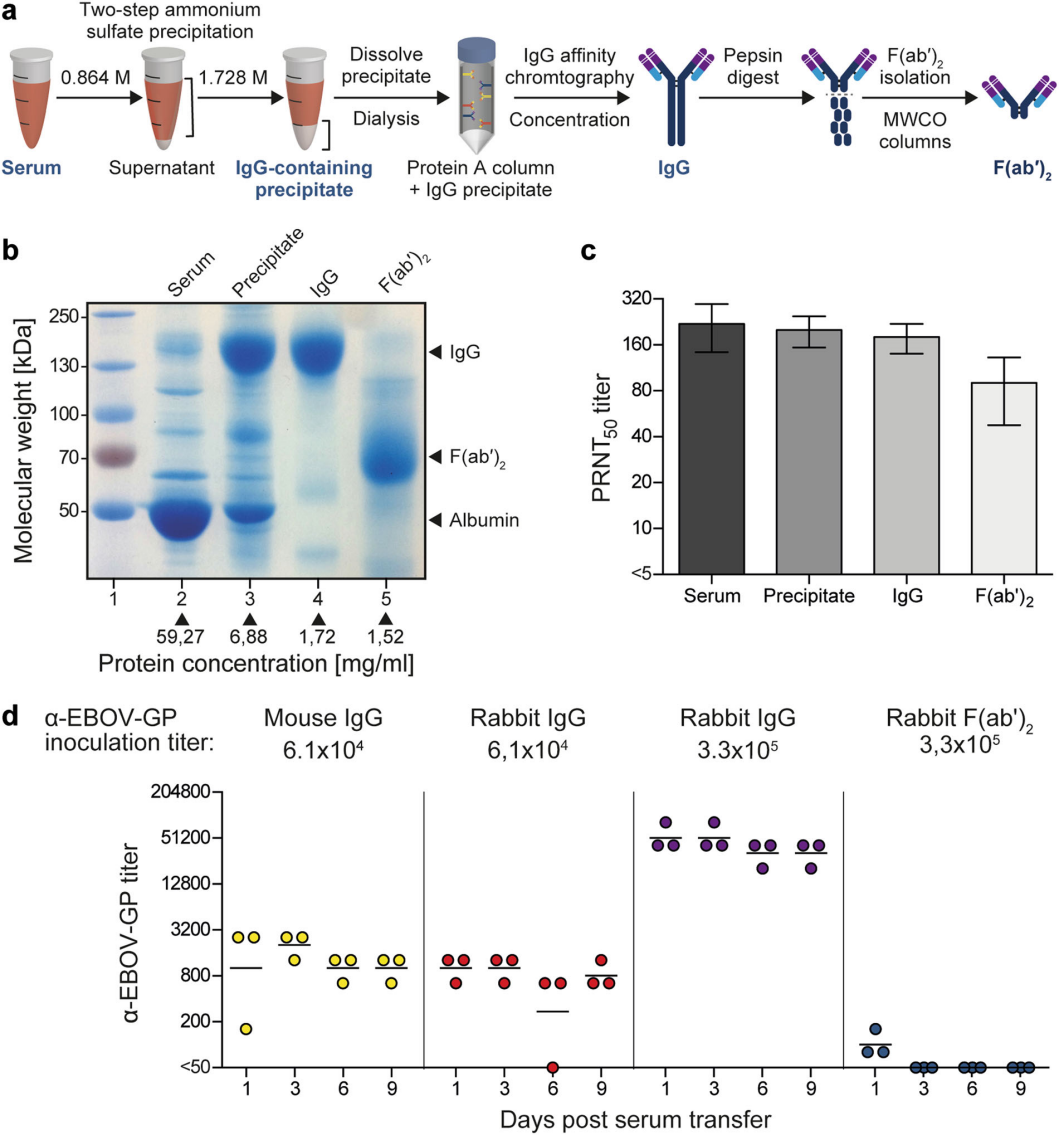
Purification and characterization of IgG and F(ab′)2 preparations. Antiserum from rabbits immunized three times with VLP-adjuvant combination was collected 1 week after the final boost. **a** Overview of the purification process showing two-step precipitation with 20 and 40% ammonium sulfate saturation, buffer exchange, and desalting, followed by IgG isolation using protein A affinity chromatography, and F(ab′)_2_ preparation by pepsin digest. Intermediates and final preparations were resuspended at an initial volume or appropriately concentrated. **b** Protein analysis of purification intermediates. Ten micrograms of total protein from each preparation was separated by non-reducing SDS-PAGE and stained with Coomassie blue. Lane 1: initial antiserum; lane 2: resuspended proteins following 20–40% precipitation; lane 3: elution of protein A affinity chromatography; and lane 4: IgG after pepsin digest. Total protein concentrations assessed by BCA assay are shown below SDS-PAGE. All blots were derived from the same experiment and were processed in parallel. **c** Neutralizing antibody response against VSVΔG/EBOV-GP of initial antiserum (*n* = 4), intermediate after precipitation (*n* = 4), affinity-purified IgG (*n* = 4), and F(ab′)_2_ preparation of affinity-purified IgG (*n* = 2) was assessed by the 50% serum neutralization capacity (PRNT_50_). Bars represent the mean of each group, and error bars indicate the standard error of the mean. **d** Mice received single doses of either purified homologous mouse IgG or heterologous rabbit IgG or F(ab′)_2_ preparation by i.p. injection. Detection of α-EBOV-GP antibodies is reported as the reciprocal serum endpoint titers with the use of VSVΔG/EBOV-GP in an IPMA assay. Symbols represent single animals and the corresponding lines for each group indicate the geometric mean (*n* = 3). The *Y*-axis begins at the detection limit of the respective assay

Acute and delayed adverse reactions to antisera, such as anaphylactic shock and serum sickness, are caused by the immunogenicity of xenogenic serum proteins or complement activation by the Fc portion of whole IgG preparations, respectively.^44^ To minimize the risk of unwanted side effects, we established a multi-step purification and concentration procedure using sera from EBOV VLP-immunized rabbits. First, serum proteins were precipitated in a two-step precipitation using 0.864 and 1.728 M ammonium sulfate followed by IgG affinity chromatography using Protein A. Next, IgG was modified by a pepsin digestion to remove the Fc portion to generate F(ab′)_2_ preparations (Fig. 4a). To accurately compare antibody titers over the course of the purification process, titers are expressed relative to the same volume for all samples. The purification and concentration is illustrated on a Coomassie-stained non-reducing SDS-PAGE gel loaded with the same protein concentration of initial serum, purification intermediates after precipitation, and the IgG and F(ab′)_2_ preparations (Fig. 4b). After ammonium sulfate precipitation, more than 90%, and even after subsequent IgG affinity purification more than 80% of neutralization activity were recovered (Fig. 4c). Pepsin digest led to around 50% loss of neutralization activity in F(ab′)_2_ preparations compared to unmodified serum, indicating that the multi-step purification process results in high yield purified IgG preparations with only little loss of functional antibody titers, and F(ab′)_2_ fragmentation with at least 50% recovery of functional activity.

### Comparable in vivo stability of homologous and xenogenic antibodies in mice

Antibody half-life is an important efficacy determinant of passive immune therapy. To assess the antibody stability in a different species, mice were treated intraperitoneally (i.p.) with purified homologous mouse IgG, or heterologous rabbit IgG preparations. Doses were calculated based on total anti-EBOV-GP antibody titers (Fig. 4d). The antibody levels in recipient mice remained stable over 9 days after injection regardless of the origin of the IgG, and treatment with a higher IgG dose resulted in increased detectable titers in the recipients (Fig. 4d).

Since F(ab′)_2_ fragments reduce the incidence of adverse events due to the lack interaction with Fc receptors, they are increasingly used to treat snake envenomation.^45^ However, they are associated with a shorter half-life, which may have a greater impact in the context of an amplifying virus than a single dose of venom.^29^ To determine the stability of the heterologous rabbit F(ab′)_2_ preparation, we injected mice intraperitoneally with different doses. In contrast to IgG, F(ab′)_2_ fragments were only detected on the first day after transfer, even if the higher dose was used (Fig. 4d), indicating that the longer half-life of IgG combined with its potential side effects may have to be weighed against the lower stability and reduced activity of F(ab′)_2_ fragments.

## Discussion

The unprecedented magnitude of the Western African EBOV outbreak resulted in the deployment of novel vaccines and therapies that were already in advanced stages of clinical development.^46^ However, for many other emerging viruses, development is not that far advanced, so there is a need for treatment options that can be rapidly produced in clinically relevant quality and quantities. Here we explored the potential of generating animal-origin hyperimmune sera in a 2-month time frame using EBOV and NiV as model antigens. We found that adjuvanted VLPs were more effective than MVA-based or VSV-based expression vectors in inducing high-titer functional antibodies after repeated immunizations, reaching levels above the putative protective threshold for the respective pathogen. An optimized purification protocol combining ammonium sulfate precipitation followed by protein A affinity purification yielded concentrated polyclonal IgG with maintained functional activity and in vivo stability, indicating that this approach may be feasible in emergency situations.

Monoclonal antibodies that neutralize EBOV, such as KZ52, are protective in some small animal models,^47^ but often fail to provide protection in more relevant NHP models.^48^ The success of the ZMapp monoclonal antibody cocktail demonstrated that targeting more than one antigenic site, including some non-neutralizing epitopes, improves efficacy.^49^ Polyclonal antisera have the advantage of inherently targeting multiple neutralizing and non-neutralizing epitopes of a given antigen, thereby reducing the risk of escape mutant emergence, another known limitation of monoclonal antibody therapies.^50,51^ This broader specificity is also a unique asset at early stages of an outbreak when the pathogen is not yet fully characterized, since cross-reactivity of already available antisera against related strains can be quickly ascertained and may provide an immediate treatment option.

Even though immunization of horses with inactivated EBOV failed to produce effective therapeutic antisera,^28^ more recent approaches using adjuvanted recombinant EBOV-GP ectodomain for immunization of sheep led to antisera that were effective as post-exposure treatment of rodents.^52,53^ The production of fully human polyclonal antibodies by immunization of transchromosomal cattle with EBOV-GP nanoparticles constitutes a further important step,^54^ but may be limited by availability of these transgenic animals. By comparing the replication-competent recombinant VSV and the replication-incompetent MVA vaccine platforms with adjuvanted VLPs, we show that even though de novo antigen expression was associated with an earlier development of total anti-EBOV antibodies, functional titers increased over the course of the immunizations for all candidates, with adjuvanted VLPs resulting in induction of similar final titers as VSVΔG/EBOV-GP. This indicates that not de novo antigen expression but repeated exposure to the antigen in its native conformation is required for the induction of high functional antibody titers.

The strong neutralizing antibody responses seen after immunization with adjuvanted VLPs not only against EBOV but also NiV demonstrates the potential of this approach to be extended to other emerging pathogens. The determinants of VLP formation in other virus families such as flaviviruses,^55^ coronaviruses,^56^ and orthomyxoviruses^57^ are known, and medium-scale to large-scale transient transfection processes are increasingly used for customized antigen or protein production.^58^ This could conceivably form the basis of a “plug-and-play” system where antigens from emerging viruses are either synthesized or cloned, then expressed and purified using such production processes, and used for immunization of serum-producing animals. The regulatory requirements regarding the quality and safety of antigens and adjuvants used for the production of antisera differ considerably from vaccines,^59,60^ as they are not applied directly to patients. Since the manufacturing of therapeutic antisera is antigen-independent, existing processes and facilities could be used with little or no further adaptation to produce such pathogen-specific antisera in an emergency situation. Furthermore, for pathogens with the potential of high public health impact, licensing under the Food Drug Administration “Animal Rule” or the EMA “Orphan Medicines” designation and subsequent stockpiling could be envisaged.^61,62^

VLPs have long been licensed as hepatitis B or human papillomavirus vaccines,^63^ and they are among the candidate platforms for emerging infections.^64^ For the generation of therapeutic antisera, VLPs combine several advantages: they are non-infectious and can thus be handled without biosafety concerns, they can be adjuvanted, they are boostable, and they can be produced in a variety of cell lines.^65,66^ For EBOV, the combination of a non-neutralizing antibody targeting the glycan cap makes the monoclonal antibody cocktail ZMapp more potent,^49^ indicating that the native glycosylation profile could be one crucial factor in the induction of effective polyclonal antibodies. In general, mammalian expression systems are able to produce post-translational modifications (PTMs), but only human cell lines have the advantage of producing complex proteins with fully human PTMs that are similar to those naturally synthetized in humans.^67^ A recent report demonstrates the efficacy of polyclonal antisera generated by immunization with VLPs produced in an insect cell expression system.^29^ However, differences in *N-*glycosylation and *O-*glycosylation profile between human and insect cell derived filovirus GPs were reported,^68^ and may affect the quality of the antisera, and limit the application of this system to other pathogens.

After multiple-step purification we observed similar in vivo stability for xenogenic rabbit IgG and homologous mouse IgG preparations in recipient mice, while rabbit F(ab′)_2_ was only detectable for 1 day after transfer. This short half-life of F(ab′)_2_ preparations was also observed in earlier studies where a single dose of antisera resulted in better protection over a longer period compared to repeated treatment with F(ab′)_2_ fragments.^29^ Until processes to stabilize F(ab′)_2_ preparations have been developed, full IgG with its known and controllable risks for adverse events may be a valuable addition to the toolbox against newly emerging infectious diseases.

## Materials and methods

### Cells and viruses

Vero E6 cells (ATCC CRL-1586), MDCK cells (ATCC CCL-34) and HEK-293 cells (ATCC CRL-1573) were maintained at 37 °C and 5% CO_2_ in Dulbecco’s modified Eagle’s medium (DMEM, Sigma-Aldrich) supplemented with 5% (v/v) fetal bovine serum (FBS, Invitrogen) and 200 mM l-glutamine (Sigma-Aldrich).

The recombinant vesicular stomatitis expressing the GP derived from Zaire EBOV isolate Kikwit in place of the original VSV GP (VSVΔG/EBOV-GP)^34^ as well as the Zaire EBOV strains Makona and Mayinga, and the NiV strain Malaysia were grown on Vero E6 cells. The viral titers of the Zaire EBOV and NiV strains were determined by limited dilution, using cytopathic effect (CPE) as read out. VSVΔG/EBOV-GP was concentrated by centrifugation for 1 h at 100,000 × *g* and 4 °C through a 20% (w/v) sucrose cushion and resuspended in DMEM or in phosphate-buffered saline (PBS) for immunizations. Viral titers were determined by plaque assay using Avicel RC-591 overlay (FMC Corporation). Briefly, 200 µl/well of 10-fold serial virus dilutions in DMEM were added to Vero E6 cells in 12-well tissue culture plates. For the overlay, 3% Avicel in PBS was mixed with an equal volume of DMEM with 5% FBS. After incubation for 1 h at room temperature with gentle agitation, 2 ml/well of 1.5% Avicel overlay was added, and plates were incubated at 37 °C for 48 h. For visualization of the plaques, 1 ml/well PBS containing 10% crystal violet, 12.5% paraformaldehyde (PFA), and 25% methanol was added and incubated for 30 min at room temperature, and titers were expressed as plaque-forming units (PFUs).

The MVA vector vaccines delivering either the GP from Zaire EBOV strain Guéckédou 2014 alone (MVA/EBOV-GP) or together with the EBOV-VP40 matrix protein (MVA/EBOV-VP40/GP) were produced by virus amplification in secondary chicken embryo fibroblasts, purified by ultracentrifugation through sucrose, and reconstituted in Tris-buffered saline (pH 7.4). The vaccine preparations were stored at −80 °C before use. All quality control experiments were essentially performed as described previously.^69^ Vaccine preparations were titrated by plaque assay using MVA-specific immunostaining, and titers were indicated in focus-forming units (FFUs).

### Expression plasmids and VLP production

The construction of mammalian expression plasmids encoding the VP40 matrix protein (pCAGGS-EBOV/VP40) or the GP (pCAGGS-EBOV/GP) of the Zaire EBOV strain Mayinga have been described previously.^70,71^ Expression plasmids encoding the matrix protein M (pCG-NiV/M and pCG-NiV/M_myc_), the GP for fusion F (pCG-NiV/F and pCG-NiV/F_HA_) or attachment G (pCG-NiV/G and pCG-NiV/G_FLAG_) of the NiV strain Malaysia have also been reported.^42^ For production of EBOV VLPs, HEK-293 cells at approximately 80% confluency were seeded in 175 cm^2^ tissue culture flasks and co-transfected with 20 µg of each of the plasmids mixed with four volumes of 18 mM polyethylenimine reagent (Sigma-Aldrich).^70^ For production of NiV VLPs, cells were transfected with 20 µg of plasmid DNA encoding for the M or M_myc_ protein and 20 µg of plasmid DNA encoding for either the F, F_HA_, G, or G_FLAG_ proteins. Transfection mixtures were prepared in DMEM without additives. After incubation at 37 °C for 12 h, the medium was changed, and the cells were incubated for another 48 h. Culture supernatants were harvested and clarified by centrifugation at 2000 × *g* for 10 min at 4 °C. VLPs were then pelleted by ultracentrifugation through a 20% (w/v) sucrose cushion for 3 h for EBOV VLPs or 2 h for NiV VLPs at 100,000 x *g* and 4 °C, and pellets were resuspended in PBS and stored at 4 °C.

### Quantification of total antibodies

Total antibody responses were determined by immunoperoxidase monolayer assay (IPMA). Briefly, for determination of EBOV-GP-specific antibodies 5 × 10^5^ Vero E6 cells were seeded in 96-well plates and infected with 100 PFU VSVΔG/EBOV-GP. For determination of NiV-F-specific and NiV-G-specific antibody responses, MDCK cells were transfected with 20 µg DNA of either pCG-NiV/F_HA_ or pCG-NiV/G_FLAG_ plasmids. Cells were incubated for 48 h at 32 °C before fixation with 2% PFA. Serial 2-fold dilutions of antisera were added to the fixed cells and incubated for 2 h at room temperature, followed by incubation with horseradish peroxidase (HRP)-coupled secondary antibody, and visualized by staining with 3-amino-9-ethylcarbazole (Sigma-Aldrich). Total antibody titers were calculated as geometric mean of the reciprocal of the last serum dilution at which positive staining was detected.

Total antibody responses against whole EBOV virus were determined by ELISA as described previously.^37,38^ Briefly, particles of the Zaire EBOV isolate Makona were purified and concentrated by ultracentrifugation through a 20% sucrose cushion, and the pellet was resuspended in PBS containing 1% SDS and inactivated by boiling for 10 min. ELISA plates were then coated with inactivated Zaire EBOV isolate Makona, and rabbit antisera were added at the respective dilution and detected by a polyclonal HRP-coupled secondary antibody. After staining with TMB (KPL Inc.) and addition of the stop solution (KPL Inc.), the OD was measured at 450–630 nm. Each sample was analyzed in duplicate and the mean OD values were corrected by subtracting mock OD values from values obtained by incubating the same serum with EBOV antigen. For endpoint titrations, serial 2-fold serum dilutions were added to coated ELISA plates. Each sample was analyzed in duplicate and the mean OD values were corrected as described above. The cut-off was calculated as the mean plus standard deviation (SD) multiplied with 10 (mean ± SD × 10) of negative rabbit sera and the total antibody titers were expressed as the reciprocal of the highest serum dilution yielding an OD value greater than the cut-off OD of 0.120.

### Quantification of neutralizing antibodies

To measure neutralizing antibodies, a VSVΔG/EBOV-GP-based PRNT assay or a virus neutralization assay with Zaire EBOV isolate Mayinga was used. For PRNT, 2-fold serial dilutions of heat-inactivated antisera in DMEM were mixed with the appropriate volume of 10^3^ PFU/ml VSVΔG/EBOV-GP to result in a final concentration of 10^2^ PFU/well. The mixture was incubated at room temperature for 30 min, and then 200 µl/well was added to confluent 12-well dishes of Vero E6 cells. After incubation for 1 h at room temperature with gentle agitation, the inoculum was removed and replaced with 2 ml 1.5% Avicel overlay. Plates were incubated at 37 °C and for 48 h, and plaques were visualized using crystal violet staining. The PRNT_50_ titer was calculated by averaging the reciprocal of the highest serum dilution reducing the number of plaques by 50% relative to the average number of plaques in positive control wells.

The virus neutralization assay for EBOV was performed as described previously.^38^ Briefly, serial 2-fold dilutions of heat-inactivated rabbit antisera preparations were incubated with 10^2^ 50% tissue culture infection doses (TCID_50_) of Zaire EBOV isolate Mayinga, and then added to Vero E6 cells. The CPE was evaluated after 7 days and titers are expressed as the geometric mean of the reciprocal of the last serum dilution at which CPE was observed.

For the virus neutralization assay of NiV antisera 2-fold serial dilutions of the rabbit sera in 96-well plates were mixed with 10^2^ TCID_50_ NiV strain Malaysia. The virus and serum dilutions were incubated for 1 h at 37 °C, after which Vero E6 cells were added to each well. The CPE was assessed after 6 days, and neutralizing titers are reported as the geometric mean of four replicates.

All experiments with infectious EBOV or NiV were performed in the BSL4 facility of the Institute of Virology, Philipps-University Marburg, in compliance with German regulations.

### Immunizations and serum transfer study

All animal experiments were carried out in compliance with the regulations of German animal protection laws and authorized by the Regierungspräsidium Darmstadt, Germany. Balb/c mice were purchased from Janvier S.A.S. and New Zealand White rabbits from Charles River Germany.

To generate mouse antisera, groups of 6-week-old Balb/c mice were immunized intramuscularly with either 2 × 10^5^ PFU of VSVΔG/EBOV-GP, 1.5 × 10^8^ PFU of either MVA/EBOV-VP40/GP or MVA/EBOV-GP, or 10 µg of EBOV VLPs mixed in a 1:1 ratio (v/v) with either Sigma Adjuvant System (0.5 mg monophosphoryl lipid A (detoxified endotoxin) from *Salmonella minnesota* and 0.5 mg synthetic trehalose dicorynomycolate in 2% squalene-Tween-80-water, Sigma), or TiterMax Gold (block polymer CRL-8300, squalene, and sorbitan monooleate, Sigma), or 2% Alhydrogel (Invivogen). All animals were boosted two and 4 weeks after the first immunization. Three mice from each immunization approach were sacrificed at 0, 14, 28, and 42 days post-immunization and blood was collected by cardiac puncture.

For generation of rabbit EBOV antisera, New Zealand White rabbits were immunized intramuscularly with either 1 × 10^8^ PFU of VSVΔG/EBOV-GP, 5 × 10^9^ PFU MVA/EBOV-GP, or 300 µg of EBOV VLPs mixed in a 1:1 ratio (v/v) with Sigma Adjuvant System, and boosted 2 and 4 weeks later. Blood samples were collected from the marginal ear vein on days 0, 14, and 28, and animals were sacrificed 35 days post-immunization by exsanguination. For generation of NiV-F or NiV-G antisera, New Zealand White rabbits were immunized intramuscularly with 300 µg of NiV VLPs containing either NiV-M/F or NiV-M/G mixed in a 1:1 ratio (v/v) with Sigma Adjuvant System, and boosted 3 and 5 weeks later. Blood samples were collected from the marginal ear vein on days 0, 21, and 35, and animals were sacrificed 49 days post-immunization by exsanguination.

To test the stability of the respective antisera in a xenogenic transfer model, 6-week-old Balb/c mice were administered 200 µl of purified mouse or rabbit IgG, or rabbit F(ab′)_2_ fragment by intraperitoneal injection. Three mice from each antiserum transfer group were sacrificed at days 1, 3, 6, and 9 after transfer and blood was collected by exsanguination. The rabbit sera were tested for total antibodies against EBOV-GP by IPMA.

### Antisera purification and fragmentation

IgG was purified from rabbit antisera using a multi-step process. First, animal sera were treated with 0.864 M (20% saturation) ammonium sulfate (Pierce) at 4 °C for 6 h to remove serum proteins that are less soluble than IgG. Precipitated proteins were removed by centrifugation at 3000 × *g* for 20 min at 4 °C. Next, the ammonium sulfate concentration of the supernatant was adjusted to 1.944 M (45% saturation) to remove serum proteins that are more soluble than IgG. After centrifugation, the supernatant was removed and the IgG-containing precipitate was dissolved in the original volume of the serum sample using 0.1 M Tris-HCl. The dissolved precipitate was dialyzed against Tris-buffered saline through a 20 kDa molecular weight cut-off (MWCO) cassettes (Thermo Scientific) before IgG was purified by affinity chromatography using protein A columns (Vivapure Kit, Sartorius) according to the manufacturer’s recommendations with some modifications. Briefly, 5 ml precipitated sample was mixed with two volumes of 1.5 M glycine/NaOH, 3 M NaCl, pH 9.0, binding buffer and centrifuged at 150 × *g* for 30 min at room temperature to let the IgG bind to protein A. This step was repeated with the flow through before the column was washed eight times with binding buffer and centrifuged at 500 × *g* for 3 min at room temperature. IgG was eluted in 10 ml 0.2 M glycine/HCl elution buffer with pH 2.5 immediately into 1.3 ml previously prepared 1 M Tris-HCl, pH 9.0, neutralization buffer to bring the samples’ pH to approximately 7.5. For concentrating to the original volume and buffer exchange of the IgG sample, 50 kDa MWCO centrifugal column (Vivaspin, Sartorius) were used according to the manufacturer’s recommendations. Concentrated IgG was dissolved in PBS for immunization, or 20 mM sodium acetate buffer, pH 4 for F(ab′)_2_ preparation. To generate F(ab′)_2_ fragments, purified IgG in acetate buffer was mixed with pepsin (>2500 U/mg, Sigma) in a 40:1 ratio and incubated at 37 °C for 6 h. The digestion was stopped with the addition of Tris base (pH 11.24) to adjust the final pH to 7.4. F(ab′)_2_ fragments were separated from undigested IgG by separation through a 100 kDa MWCO centrifugal concentrator and from remaining peptides and pepsin by separation through a 50 kDa MWCO centrifugal concentrator before dissolving in PBS.

### SDS-PAGE and Western blot analysis

The protein concentration of purified VLPs was determined by Bradford assay (Bio-Rad), and the BCA assay (Thermo Fisher) was used for samples of the serum purification process. For verification of sample quality and purity, EBOV VLP samples containing 10 µg total protein or NiV VLP samples containing 3 µg total protein were separated by 10% reducing SDS-PAGE and antiserum samples containing 10 µg total protein were separated by 8% non-reducing SDS-PAGE and stained with Coomassie blue (Fisher Bioreagents) or transferred to polyvinylidene difluoride (PVDF) membranes (Millipore) for Western blot analysis. Blots were stained with a polyclonal goat antiserum against EBOV virus^72^ at a working solution of 1:1000 or monoclonal antibodies against FLAG-tag, HA-tag, or c-myc-tag (all Sigma) at the recommended dilution to detect the respective epitope-tagged NiV proteins.

### Statistical analysis

Statistical analyses were performed using GraphPad Prism 6 on log-transformed data using multiple comparison analysis of the final mouse sera in one-way analysis of variance with Tukey's post-test. Significance was considered at *p* values ≤0.05.

## Electronic supplementary material

Supplementary Information

## Data Availability

The data reported in this paper are available from the corresponding author upon request.

## Electronic supplementary material

**Supplementary information** accompanies the paper on the *npj Vaccines* website (10.1038/s41541-018-0082-4).

## References

[1] Agua-Agum J (2016). After Ebola in West Africa–unpredictable risks, preventable epidemics. N. Engl. J. Med..

[2] Uyeki TM (2016). Clinical management of Ebola virus disease in the United States and Europe. N. Engl. J. Med..

[3] World Health Organization. (1978). Report of an International Commission. Ebola haemorrhagic fever in Zaire, 1976. Bull. World Health Organ..

[4] Khan AS (1999). The reemergence of Ebola hemorrhagic fever, Democratic Republic of the Congo, 1995. Commission de Lutte contre les Epidemies a Kikwit. J. Infect. Dis..

[5] Lo MK (2012). Characterization of Nipah virus from outbreaks in Bangladesh, 2008–2010. Emerg. Infect. Dis..

[6] Mahalingam S (2012). Hendra virus: an emerging paramyxovirus in Australia. Lancet Infect. Dis..

[7] Paul, L. Nipah virus in Kerala: a deadly zoonosis. *Clin. Microbiol. Infect.*10.1016/j.cmi.2018.06.017 (2018). 10.1016/j.cmi.2018.06.017 29935330

[8] Chua KB (1999). Fatal encephalitis due to Nipah virus among pig-farmers in Malaysia. Lancet.

[9] Chua KB (2000). Nipah virus: a recently emergent deadly paramyxovirus. Science.

[10] World Health Organization. *Statement. Ethical Considerations for Use of Unregistered Interventions for Ebola Virus Disease (EDV): Summary of the Panel Discussion* http://www.who.int/mediacentre/news/statements/2014/ebola-ethical-review-summary/en/ (2014).

[11] Goh KJ (2000). Clinical features of Nipah virus encephalitis among pig farmers in Malaysia. N. Engl. J. Med..

[12] Stille, W. & Böhle, E. in *Marburg Virus Disease* (eds Martini, G. A. & Siegert, R.) 10–18 (Springer, Berlin, Heidelberg, 1971).

[13] Todorovitch, K., Mocitch, M. & Klašnja, R. in *Marburg Virus Disease* (eds Martini, G. A. & Siegert, R.) 19–23 (Springer, Berlin, Heidelberg, 1971).

[14] Mupapa K (1999). Treatment of Ebola hemorrhagic fever with blood transfusions from convalescent patients. International Scientific and Technical Committee. J. Infect. Dis..

[15] Jahrling PB, Geisbert JB, Swearengen JR, Larsen T, Geisbert TW (2007). Ebola hemorrhagic fever: evaluation of passive immunotherapy in nonhuman primates. J. Infect. Dis..

[16] Griensven Jv (2016). Evaluation of convalescent plasma for Ebola virus disease in Guinea. N. Engl. J. Med..

[17] Dye JM (2012). Postexposure antibody prophylaxis protects nonhuman primates from filovirus disease. Proc. Natl. Acad. Sci. USA.

[18] Satterfield BA, Dawes BE, Milligan GN (2016). Status of vaccine research and development of vaccines for Nipah virus. Vaccine.

[19] Guillaume V (2004). Nipah virus: vaccination and passive protection studies in a Hamster Model. J. Virol..

[20] DeBuysscher BL, Scott D, Marzi A, Prescott J, Feldmann H (2014). Single-dose live-attenuated Nipah virus vaccines confer complete protection by eliciting antibodies directed against surface glycoproteins. Vaccine.

[21] Guillaume V (2006). Antibody prophylaxis and therapy against Nipah virus infection in hamsters. J. Virol..

[22] Bossart KN (2009). A neutralizing human monoclonal antibody protects against lethal disease in a new ferret model of acute Nipah virus infection. PLoS Pathog..

[23] Center for Disease Control and Prevention. *Use of a Reduced (4-Dose) Vaccine Schedule for Postexpousre Prophylaxis to Prevent Human Rabies: Recommendations of the Advisory Committee on Immunization Practice* http://www.cdc.gov/mmwr/pdf/rr/rr5902.pdf (2010). 10.1016/j.annemergmed.2010.05.020 20648715

[24] Gupta PS, Kapoor R, Goyal S, Batra VK, Jain BK (1980). Intrathecal human tetanus immunoglobulin in early tetanus. Lancet.

[25] Walsh JJ (2007). A case of naturally acquired inhalation anthrax: clinical care and analyses of anti-protective antigen immunoglobulin G and lethal factor. Clin. Infect. Dis..

[26] Keller MA, Stiehm ER (2000). Passive immunity in prevention and treatment of infectious diseases. Clin. Microbiol. Rev..

[27] Boyer L (2013). Safety of intravenous equine F(ab′)2: insights following clinical trials involving 1534 recipients of scorpion antivenom. Toxicon.

[28] Jahrling PB (1996). Passive immunization of Ebola virus-infected cynomolgus monkeys with immunoglobulin from hyperimmune horses. Arch. Virol. Suppl..

[29] Zheng X (2016). Treatment with hyperimmune equine immunoglobulin or immunoglobulin fragments completely protects rodents from Ebola virus infection. Sci. Rep..

[30] Noad R, Roy P (2003). Virus-like particles as immunogens. Trends Microbiol..

[31] Wahl-Jensen V (2005). Role of Ebola virus secreted glycoproteins and virus-like particles in activation of human macrophages. J. Virol..

[32] Aleksandrowicz P (2011). Ebola virus enters host cells by macropinocytosis and clathrin-mediated endocytosis. J. Infect. Dis..

[33] Jasenosky LD, Neumann G, Lukashevich I, Kawaoka Y (2001). Ebola virus VP40-induced particle formation and association with the lipid bilayer. J. Virol..

[34] Geisbert TW (2008). Vesicular stomatitis virus-based Ebola vaccine is well-tolerated and protects immunocompromised nonhuman primates. PLoS Pathog..

[35] Marzi A (2011). Vesicular stomatitis virus-based Ebola vaccines with improved cross-protective efficacy. J. Infect. Dis..

[36] Marzi A (2015). VSV-EBOV rapidly protects macaques against infection with the 2014/15 Ebola virus outbreak strain. Science.

[37] Krahling V (2016). Development of an antibody capture ELISA using inactivated Ebola Zaire Makona virus. Med. Microbiol. Immunol..

[38] Agnandji ST (2016). Phase 1 trials of rVSV Ebola vaccine in Africa and Europe. N. Engl. J. Med..

[39] Mire CE (2013). Single injection recombinant vesicular stomatitis virus vaccines protect ferrets against lethal Nipah virus disease. Virol. J..

[40] Walpita P (2017). A VLP-based vaccine provides complete protection against Nipah virus challenge following multiple-dose or single-dose vaccination schedules in a hamster model. NPJ Vaccin..

[41] Zhu Z (2006). Potent neutralization of Hendra and Nipah viruses by human monoclonal antibodies. J. Virol..

[42] Sawatsky B, Bente DA, Czub M, Messling Vv (2016). Morbillivirus and henipavirus attachment protein cytoplasmic domains differently affect protein expression, fusion support and particle assembly. J. Gen. Virol..

[43] Wang LF (2001). Molecular biology of Hendra and Nipah viruses. Microbes Infect..

[44] Silva HAd, Ryan NM, Silva HJd (2016). Adverse reactions to snake antivenom, and their prevention and treatment. Br. J. Clin. Pharmacol..

[45] Dart RC, McNally J (2001). Efficacy, safety, and use of snake antivenoms in the United States. Ann. Emerg. Med..

[46] Wong G, Kobinger GP (2015). Backs against the wall: novel and existing strategies used during the 2014–2015 Ebola virus outbreak. Clin. Microbiol. Rev..

[47] Parren PWHI, Geisbert TW, Maruyama T, Jahrling PB, Burton DR (2002). Pre- and postexposure prophylaxis of Ebola virus infection in an animal model by passive transfer of a neutralizing human antibody. J. Virol..

[48] Oswald WB (2007). Neutralizing antibody fails to impact the course of Ebola virus infection in monkeys. PLoS Pathog..

[49] Davidson E (2015). Mechanism of binding to Ebola virus glycoprotein by the ZMapp, ZMAb, and MB-003 cocktail antibodies. J. Virol..

[50] Qiu X, Kobinger GP (2014). Antibody therapy for Ebola: Is the tide turning around?. Hum. Vaccin. Immunother..

[51] Kugelman JR (2015). Evaluation of the potential impact of Ebola virus genomic drift on the efficacy of sequence-based candidate therapeutics. mBio.

[52] Dowall SD (2016). Development of a cost-effective ovine polyclonal antibody-based product, EBOTAb, to treat Ebola virus infection. J. Infect. Dis..

[53] Dowall SD (2016). Post-exposure treatment of Ebola virus disease in guinea pigs using EBOTAb, an ovine antibody-based therapeutic. Sci. Rep..

[54] Dye JM (2016). Production of potent fully human polyclonal antibodies against Ebola Zaire virus in transchromosomal cattle. Sci. Rep..

[55] Schalich J (1996). Recombinant subviral particles from tick-borne encephalitis virus are fusogenic and provide a model system for studying flavivirus envelope glycoprotein functions. J. Virol..

[56] Mortola E, Roy P (2004). Efficient assembly and release of SARS coronavirus-like particles by a heterologous expression system. FEBS Lett..

[57] Latham T, Galarza JM (2001). Formation of wild-type and chimeric influenza virus-like particles following simultaneous expression of only four structural proteins. J. Virol..

[58] Cervera L, Kamen AA (2018). Large-scale transient transfection of suspension mammalian cells for VLP production. Methods Mol. Biol..

[59] European Medicines Agency. *Production and Quality Control of Animal Immunoglobins and Immunosera for Human Use: EMA/CHMP/BWP/3354/1999.* http://www.ema.europa.eu/docs/en_GB/document_library/Scientific_guideline/2016/08/WC500211639.pdf (2016).

[60] Food and Drug Administration. *Guidance for Industry. For the Submission of Chemistry, Manufacturing and Controls and Establishment Description Information for Human Plasma-Derived Biological Products, Animal Plasma or Serum-Derived Products.* https://www.fda.gov/downloads/BiologicsBloodVaccines/GuidanceComplianceRegulatoryInformation/Guidances/Blood/UCM080825.pdf (1999).

[61] Park GD, Mitchel JT (2016). Working with the U.S. Food and Drug Administration to obtain approval of products under the Animal Rule. Ann. N. Y. Acad. Sci..

[62] Hofer MP (2018). Marketing authorisation of orphan medicines in Europe from 2000 to 2013. Drug Discov. Today.

[63] Wang JW, Roden RBS (2013). Virus-like particles for the prevention of human papillomavirus-associated malignancies. Expert. Rev. Vaccin..

[64] Boigard H (2017). Zika virus-like particle (VLP) based vaccine. PLoS Negl. Trop. Dis..

[65] Zeltins A (2013). Construction and characterization of virus-like particles: a review. Mol. Biotechnol..

[66] Kushnir N, Streatfield SJ, Yusibov V (2012). Virus-like particles as a highly efficient vaccine platform: diversity of targets and production systems and advances in clinical development. Vaccine.

[67] Dumont J, Euwart D, Mei B, Estes S, Kshirsagar R (2016). Human cell lines for biopharmaceutical manufacturing: history, status, and future perspectives. Crit. Rev. Biotechnol..

[68] Clarke EC (2017). Production and purification of filovirus glycoproteins in insect and mammalian cell lines. Sci. Rep..

[69] Kremer M (2012). Easy and efficient protocols for working with recombinant vaccinia virus MVA. Methods Mol. Biol..

[70] Hoenen T (2005). VP40 octamers are essential for Ebola virus replication. J. Virol..

[71] Mittler E, Kolesnikova L, Hartlieb B, Davey R, Becker S (2011). The cytoplasmic domain of Marburg virus GP modulates early steps of viral infection. J. Virol..

[72] Groseth A (2012). The Ebola virus glycoprotein contributes to but is not sufficient for virulence in vivo. PLoS Pathog..

