# Supplementary material for: Generation of therapeutic antisera for emerging viral infections
Source: NPJ Vaccines. 2018 Oct 5;3:42. doi: 10.1038/s41541-018-0082-4 (PMC6173733; doi:10.1038/s41541-018-0082-4)
Supplement: Supplementary file 1 — Supplementary Information [file 41541_2018_82_MOESM1_ESM.pptx]

## Slide 1
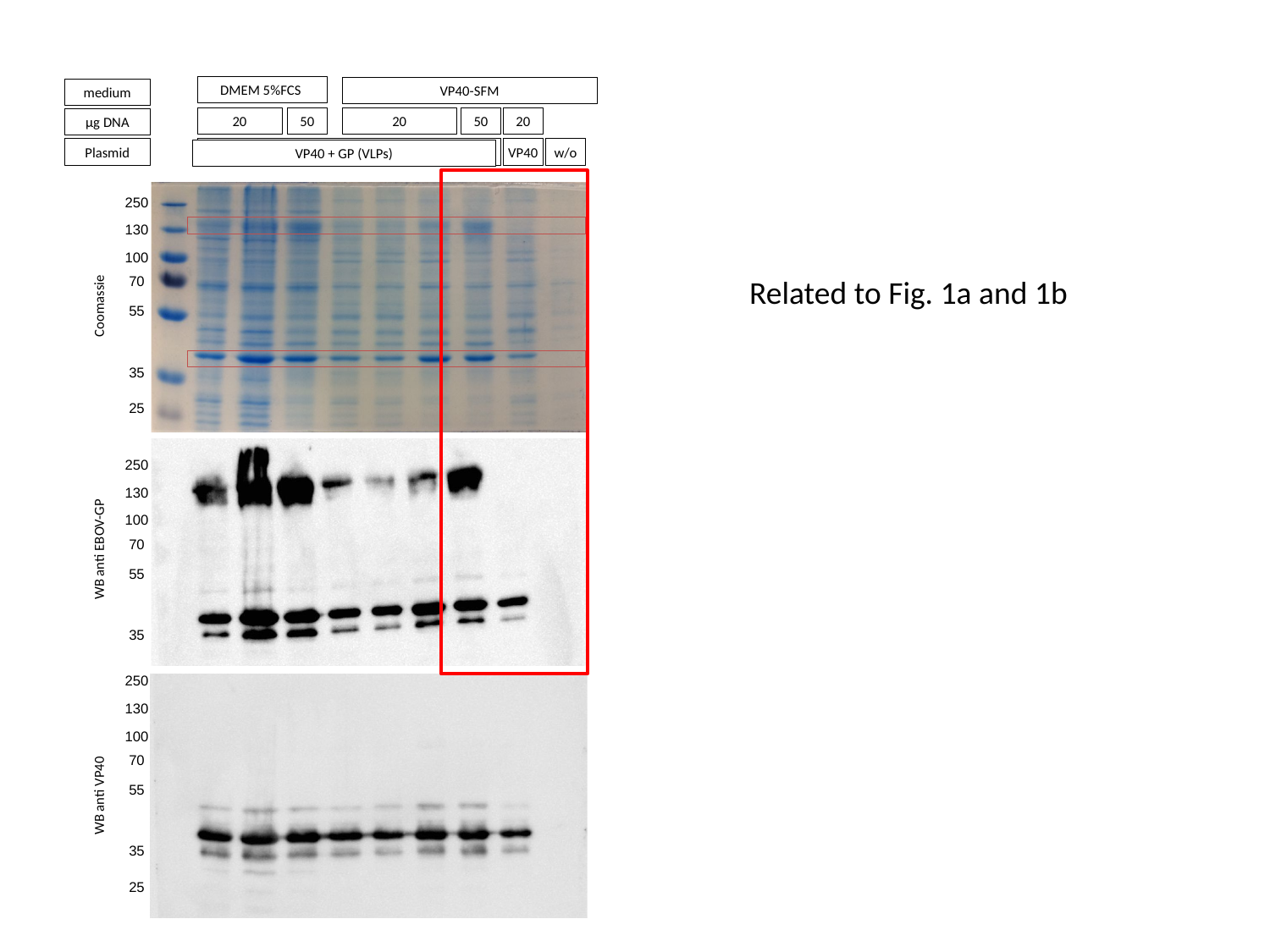

DMEM 5%FCS
VP40-SFM
medium
20
20
50
20
50
µg DNA
Plasmid
VP40 + GP (VLPs)
VP40
w/o
VP40 + GP (VLPs)
250
130
100
70
Related to Fig. 1a and 1b
Coomassie
55
35
25
250
130
100
70
55
35
WB anti EBOV-GP
250
130
100
70
55
35
25
WB anti VP40

## Slide 2
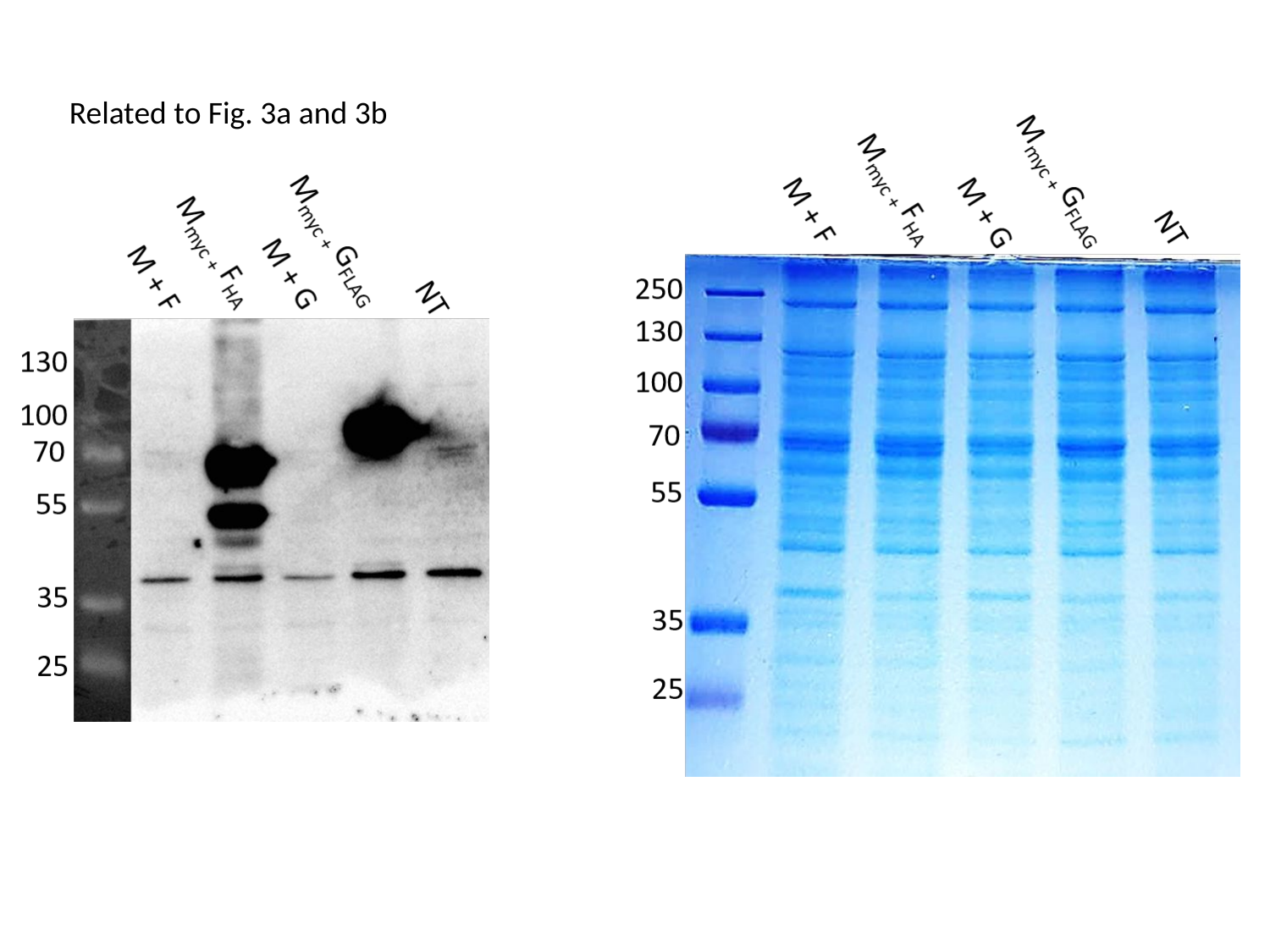

Related to Fig. 3a and 3b

## Slide 3
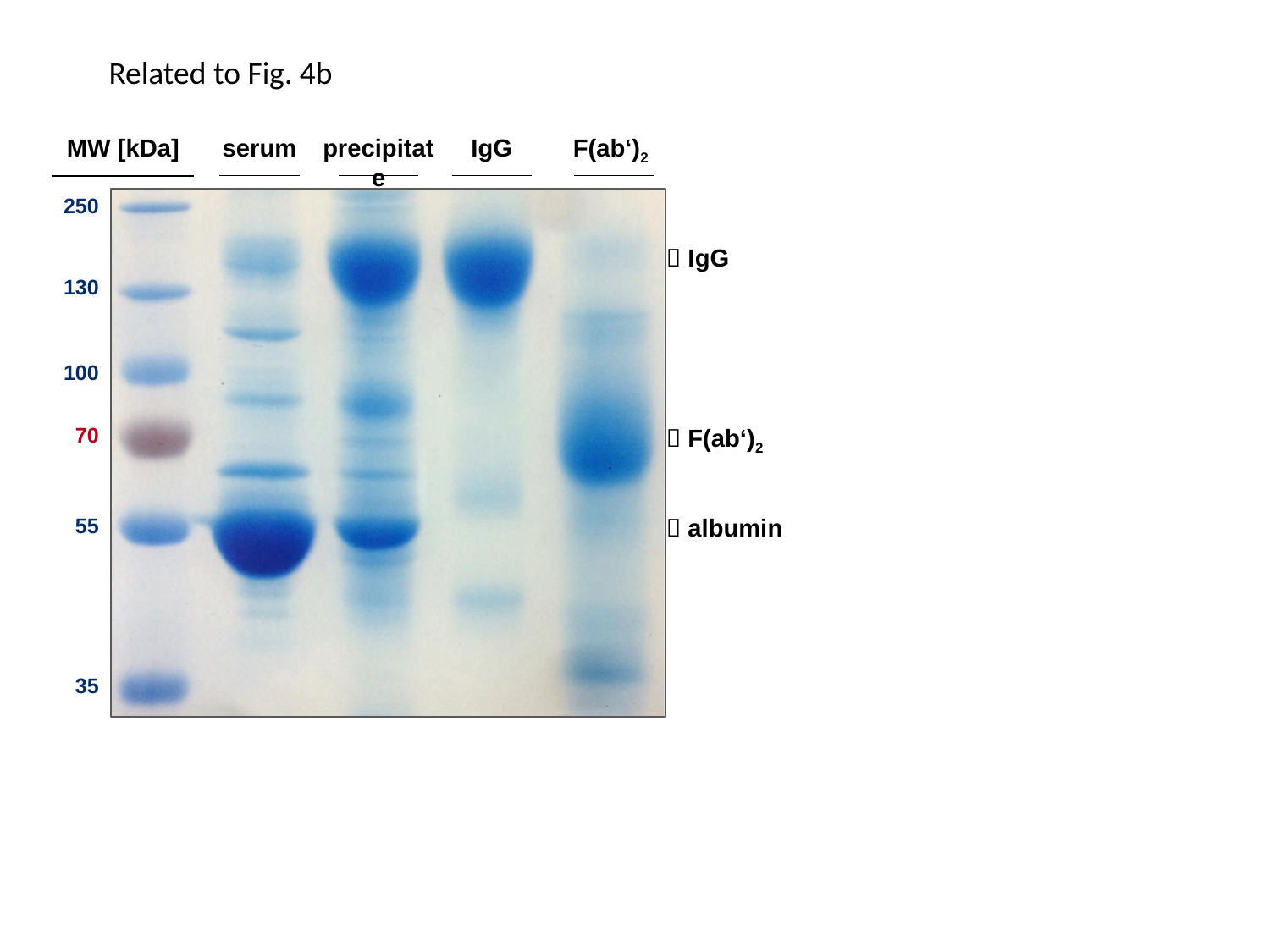

Related to Fig. 4b
MW [kDa]
serum
precipitate
IgG
F(ab‘)2
250
 IgG
130
100
70
 F(ab‘)2
55
 albumin
35
